# Two phase feature-ranking for new soil dataset for *Coxiella burnetii* persistence and classification using machine learning models

**DOI:** 10.1038/s41598-022-26956-8

**Published:** 2023-01-02

**Authors:** Fareed Ahmad, Muhammad Usman Ghani Khan, Ahsen Tahir, Muhammad Yasin Tipu, Masood Rabbani, Muhammad Zubair Shabbir

**Affiliations:** 1 Department of Computer Science, University of Engineering and Technology, Lahore, Pakistan; 2 Quality Operations Laboratory, Institute of Microbiology, University of Veterinary and Animal Sciences, Lahore, Pakistan; 3 Institute of Microbiology, University of Veterinary and Animal Sciences, Lahore, Pakistan; 4 Department of Electrical Engineering, University of Engineering and Technology, Lahore, Pakistan; 5 Department of Pathology, University of Veterinary and Animal Sciences, Lahore, Pakistan

**Keywords:** Computational biology and bioinformatics, Diagnosis, Disease prevention, Infectious diseases, Microbiology, Infectious-disease epidemiology

## Abstract

*Coxiella burnetii* (Cb) is a hardy, stealth bacterial pathogen lethal for humans and animals. Its tremendous resistance to the environment, ease of propagation, and incredibly low infectious dosage make it an attractive organism for biowarfare. Current research on the classification of Coxiella and features influencing its presence in the soil is generally confined to statistical techniques. Machine learning other than traditional approaches can help us better predict epidemiological modeling for this soil-based pathogen of public significance. We developed a two-phase feature-ranking technique for the pathogen on a new soil feature dataset. The feature ranking applies methods such as ReliefF (RLF), OneR (ONR), and correlation (CR) for the first phase and a combination of techniques utilizing weighted scores to determine the final soil attribute ranks in the second phase. Different classification methods such as Support Vector Machine (SVM), Linear Discriminant Analysis (LDA), Logistic Regression (LR), and Multi-Layer Perceptron (MLP) have been utilized for the classification of soil attribute dataset for Coxiella positive and negative soils. The feature-ranking methods established that potassium, chromium, cadmium, nitrogen, organic matter, and soluble salts are the most significant attributes. At the same time, manganese, clay, phosphorous, copper, and lead are the least contributing soil features for the prevalence of the bacteria. However, potassium is the most influential feature, and manganese is the least significant soil feature. The attribute ranking using RLF generates the most promising results among the ranking methods by generating an accuracy of 80.85% for MLP, 79.79% for LR, and 79.8% for LDA. Overall, SVM and MLP are the best-performing classifiers, where SVM yields an accuracy of 82.98% and 81.91% for attribute ranking by CR and RLF; and MLP generates an accuracy of 76.60% for ONR. Thus, machine models can help us better understand the environment, assisting in the prevalence of bacteria and decreasing the chances of false classification. Subsequently, this can assist in controlling epidemics and alleviating the devastating effect on the socio-economics of society.

## Introduction

Zoonotic infections are not simply medical curiosities but critical factors determining a community’s health^1^. These diseases can quickly spread with or without mechanical or biological vectors from animals to humans^2,3^. Over the past few decades, the outbreaks of zoonotic infections have increased, with enormous global social and economic effects^4^. For instance, the annual monetary loss due to food-borne infections in the US and Canada exceeds billions of dollars^1,5^. Various researchers estimate that 61% of all recognized contagious diseases in humans originate from animals and another 75% of re-emerging infections also spread from animals^6^. *Coxiella burnetii (Cb)* causes Q-fever, which is a primary global zoonotic disease. The “Q” originates from “query” fever, the name of the infection until its valid reason was found in the 1930s^7,8^. *C. burnetii* is a hardy, obligate intracellular bacteria that induce global zoonosis. The bacterium is listed as a category B biological agent by CDC and registered as a notifiable infection by OIE^9^. The Key reservoirs of the bacteria are domestic animals (primarily goats, sheep, cattle, etc.). However, previous studies of human Q-fever outbreaks have shown a relationship between the occurrence of the disease and small ruminants^10^. The organism is shed from birthing areas of infected animals, contaminating the surroundings, which stays infectious for a long time^11,12^. When the bacteria are in the atmosphere and not reproducing, they can survive in the dust, soil, and aerosol to form spore-like tiny cell variants, resistant to ultraviolet radiation and drying. Similarly, the bacteria can scatter over far-flung distances due to rains and blowing winds^13^. This pathogen could be acquired by humans either through aerosolized atoms from generative discharges, tissues, and atmospheric debris or direct interaction with affected animals’ urine, milk, semen, and feces^14^. Although affected animals and humans remain asymptomatic in several cases, reproductive disorders and undifferentiated febrile disease in animals have been documented^7^. Since its first recognition in Australian slaughterhouses, Q-fever has been regarded widespread and has appeared and reappeared worldwide^15^. This epidemic received global attention due to current outbreaks in European countries that affected humans and animals^16^. However, many patients with Q-fever stay undiagnosed due to the scarcity of suitable diagnostic facilities in underdeveloped nations and tend to be mistaken for other diseases, such as abortions or fevers of unknown origin^9^.

Developed nations have stringent benchmarks for managing biological materials and wastes, such as parasites, viruses, fungi, bacteria, secretions, or corpses of diseased animals^17^, which either cause or present a future risk to the health of humans and animals. However, there is a dire need for measures to manage these waste materials in third-world countries like Pakistan. The biological wastes of animals decay in the earth and propagate to far-flung areas due to rains, floods, blowing winds, etc. Detecting *C. burnetii* in the soil can help prevent Q-fever disease outbreaks. The approaches generally used for recognizing *C. burnetii* are ELISA^18^, PCR^19^, and mass spectroscopy (MS)^20^. Although these tests are expensive, we can reduce their operational cost by assessing only those specimens that are more likely to be positive for *C. burnetii*. This initial screening can be conducted by classifying soil specimens depending on their pH, moisture, type of soil, presence or absence of minerals, etc.

Conventionally, microbes are categorized by their purification, demonstration, and isolation of the presence of several microbial enzymes in them. However, DNA-based systems of recognition of microbes are recently gaining more popularity because of the speed and ease of implementing these tests. On the other hand, machine learning methodologies differ from conventional methods of microbial identification in that they use soil characteristics that maximize a pathogen’s survival in the atmosphere and predict the expected result before resorting to actual microbial isolation. First, we will give an overview of the environment suitable for the prevalence of various pathogens. Secondly, we will elaborate on various machine-learning approaches applied to similar problems in this domain.

Although the research data related to the suitable environment required for the persistence of these bacteria is limited, some researchers recommend that soil texture and heavy metals play a vital role in the persistence and survival of these pathogens. Some of these bacterial pathogens show great affinity towards salt and moisture in the environment.

Pathogens like *Francisella tularensis*, and *C. burnetii* have been isolated from soil, mud, and water contaminated by bodies of dead animals. These organisms may be capable of multiplication in these environments^8,21^. The researches reveal^22–26^ that physical and chemical factors like total soluble salt, organic matter, clay, moisture, silt, macro and micro-nutrients like carbon, phosphorous, sodium, potassium, sulfate calcium, and magnesium play an essential part in the prevalence of various pathogens like *C. burnetii*, *F. tularensis*, *Burkholderia mallei*, etc. Results also suggest that the soil reseves as a reservoir for the prevalence and further dispersion of pathogens in the environment. Generally, soil pH is essential in shaping bacterial communities in soils. Previous studies demonstrate that low pH is vital for the metabolic activity of *C. burnetii*. Results also suggest that the soil is a reservoir for the prevalence and further dispersion of pathogens in the environment. Generally, soil pH is essential in shaping bacterial communities in soils. Previous studies demonstrate that low pH is vital for the metabolic activity of *C. burnetii*^27^. Some works^8^ suggest that factors, such as soil moisture and vegetation, are relevant to the prevalence of *C. burnetii*. It is further reported^28^ that hot and dry conditions mainly help wind-borne dispersion of *C. burnetii* aerosols.

Though, there is only a little work presented for classifying pathogens in soil-related environments using machine-learning techniques, except for our previous works. In our initial work^29^, we applied artificial neural networks to classify *F. tularensis* (Ft) using the soil attribute dataset. The method attained an accuracy of 82.61% with the help of 1 hidden layer with 10 neurons. The soil attribute dataset contains 147 instances for Ft negative and positive sites. Each instance contains 21 features and a class attribute. In our next work^30^, we further improved the accuracy to 84.35%. We applied feature ranking to identify the features that are most related, e.g., clay, nitrogen, zinc, nickel, organic matter, soluble salts, silt, and those that are least related, e.g., potassium, phosphorous, iron, calcium, copper, chromium, sand towards the survival of the pathogen.

Table 1 gives an overview of various statistical and machine-learning approaches applied to assess the role of environmental features in the prevalence of different pathogens. The work focuses on feature ranking and classification utilizing various machine-learning techniques. The automatic classification of *C. burnetii*, along with identifying the most relevant features that help it prolong environmental survival, employing machine learning models can yield more reliable, accurate, and standardized results. Our work contribution can be summarized as shown below:

1. We present a novel soil attribute dataset for Coxiella positive and negative sites containing 21 soil features.
2. To the best of our knowledge, it is the first time our research has applied machine learning models instead of contemporary statistical models for understanding the behavior of *C. burnetii* in the environment.
3. Our model performs a two-phase feature ranking. Initially, attributes are ranked based on feature-ranking methods, and then a combination of techniques is applied to calculate the weighted scores to determine the final soil attribute ranks.
4. The model also compares the performance of feature-ranking algorithms and machine learning classifiers.
5. Our model performs classification and identifies the most relevant features that help prolong the pathogen’s survival in the environment with a high classification accuracy of up to 82.98%.
6. We apply 10-fold cross-validation to establish the performance of the proposed method.

**Table 1:** A comparison of Statistical and Machine learning techniques applied to assess the contribution of environmental features for the prevalence of pathogens.

Approach	Dataset	Number of Features	Statistical/ML techniques	Detail of results
2007^24^	Various types of Soil samples	4 attributes pH, C Moisture, particle-size	None	Organic carbon may favor the survival of *C. burnetii* in soil.
2009^31^	Lake water samples	3 attributes C(glucose), N (NH4Cl) and P (Na3PO4)	Wilcoxon’s rank, sum test, Welch two-sample, t-tests	High nutrient conditions were found to favor *F. tularensis*.
2011^32^	Soil, weather, vegetation samples	5 attributes pit, clay, sand, Mg, soil moisture, temperature	Mean, min, max, logistic regression, Student’s t-test	Soil moisture and vegetation help in the transmission of *C. burnetii*.
2014^33^	Three types of soil with mircobial diverstiy	6 attributes Soil moisture & texture, organic matter,Total S,N,C	Variance, mean, linear regression	Netural pH, C, N, S enhance the survival of E. coli and Salmonella
2015^34^	16 types of soil microcosms	6 attributes Soil texture, pH, phosphate, Organic C, total N and Water-holding capacity	Variance analysis	Pentachlorophenol(PCP) result in a depressing effect on soil microbial activity. However B. nivea and S. brumptii tolerate and degrade PCP in soil.
2015^23^	145 soil samples	21 attributes pH, sand, silt, clay, macro and micro nutrients	Odd ratio(OR) and T-test	Different physicochemical features contribute towards the survival of *F. tularensis*,*C. burnetii*, B. anthracis
2016^9^	94 soil samples	21 features sand,pH, silt, clay, macro and micro nutrients	Odd ratio(OR) and Logistic Regression (LR)	Organic matter, Na are positively related & calcium,potassium are negatively related to *C. burnetii*.
2017^26^	22 soil samples	18 attributes pH, sand, silt, clay, micro and macro nutrients	Odd ratio(OR), Dunnett’s T3, Tukey-Kramer	Na, moisture are positively related to B. malle.
2017^22^	145 soil samples	21 features sand, pH, silt, clay, macro and micro nutrients	T-test	Different chemical and physical features contribute towards the survival of *F. tularensis*.
2018^29^	145 soil samples	21 features sand, pH, silt, clay, micro and macro nutrients	Artificial Neural Networks	ANN Model achieved an accuracy of 82.61% for classification of *F. tularensis*.
2020^30^	145 soil samples	21 features sand, pH, silt, clay, macro and micro nutrients	ANN, SVM, LR, Random forest, Feature ranking methods	ANN Model achieved an accuracy of 84.35% for classification of *F. tularensis*. The most related features are clay, N, Zn, Ni, silt, organic matter, soluble salts, and least related features are K, P, Fe, Ca, Cu, Cr, sand.

**Figure 1:**
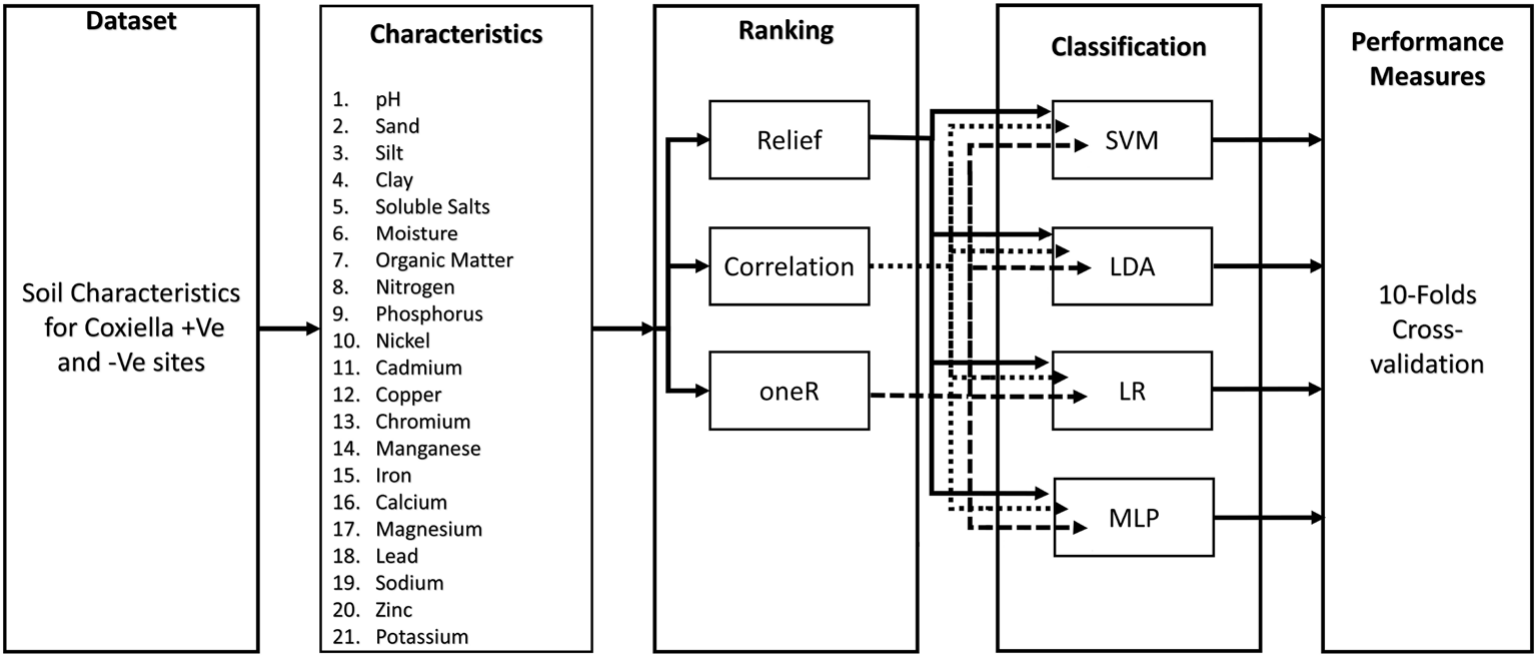
Various phases of Coxiella classification in Soil attribute dataset.

## Material and methods

The research concentrates on a comparative study of different state-of-the-art machine learning techniques employed in various fields for classification and feature ranking in a unique soil attribute dataset for Coxiella +Ve and -Ve sites. Further, we compare the performance of state-of-the-art feature ranking models and classifiers. Lastly, we propose a machine-learning model for the classification of Coxiella using soil attribute data, as exhibited in Fig. 1.

### Coxiella soil attribute dataset acquisition

Approximately 500–800 g of soil sample was taken from *C. burnetii* positive (n=47) and negative (n=47) sites using a portable electronic balance. The dataset contains 21 chemical and physical soil features, such as maximum soluble salt, organic matter, silt, clay, and micro and macro-nutrients. These physical and chemical soil features have different values, as shown in Table 2. The dataset is the property of the Institute of Microbiology, Veterinary and Animal Sciences University, Lahore, Pakistan^22^.

**Table 2:** Range of various Pysical and Chemical Soil features.

Soil attributes	Range of features
1. pH	5.9–12.2
2. Moisture (MO)	3.30–15.0%
3. Soluble Salts (SS)	0.69–5.04 mg/kg
4. Organic Matter (OM)	0.73–4.42 mg/kg
5. Clay (cy)	1.00–92.0 mg/kg
6. Sand	7.00–97.0 mg/kg
7. Silt (Si)	0.00–60.0 mg/kg
8. Nitrogen (N)	0.04–0.22 mg/kg
9. Phosphorus (P)	0.36–110.0 mg/kg
10. Magnesium (Mg)	20.37–324.4 mg/kg
11. Copper (Cu)	0.02–2.36 mg/kg
12. Chromium (Cr)	0.002–0.48 mg/kg
13. Nickel (Ni)	0.0024–14.43 mg/kg
14. Manganese (Mn)	0.09-–49.26 mg/kg
15. Cobalt (Co)	0.004–6.13 mg/kg
16. Lead (Pd)	0.22–7.60 mg/kg
17. Cadmium (Cd)	0.03–3.84 mg/kg
18. Sodium (Na)	21.1–304.9 mg/kg
19. Iron (Fe)	0.34–53.9 mg/kg
20. Calcium (Ca)	40.8–259.9 mg/kg
21. Potassium (K)	6.70–448.6 mg/kg

### Appropriate dataset for analysis

To propose an efficient and reliable machine learning model, one should select those soil features for the dataset that seem to contribute towards the prevalence and growth of *C. burnetii*. The work retrieves the most important attributes, such as pH, Moisture (MO), Soluble Salt (SS), Organic Matter (OM), Clay (cy), Sand, Silt (Si), Nitrogen (N), Phosphorus (P), Magnesium (Mg), Copper (Cu), Chromium (Cr), Nickel (Ni), Manganese (Mn), Cobalt (Co), Lead (Pb), Cadmium (Cd), Sodium (Na), Iron (Fe), Calcium (Ca), Potassium (K) for the study.

### Feature selection

In order to assemble an efficient and accurate model that would improve performance, data filtering is essential. These types of models would allow us to extract the best set of attributes. Suppose 21 input features are extracted from the soil feature dataset. In this article, ${{\textbf {X}}}_{\textrm{dn}}=[\hbox {X}_{\textrm{1n}}, \hbox {X}_{\textrm{2n}},\ldots , \hbox {X}_{\textrm{Dn}}]$ represents the feature matrix with D column vectors, and $\hbox {x}_{\textrm{dn}}$ is a certain feature value (with $\hbox {d}=1, \,2, \,3, \ldots \hbox { D}$ and $\hbox {n}=1, \,2, \,3,\ldots \hbox { N}$; being D=21 and N=94 in the dataset).

### Attribute selection models

An attribute selection model combines a search function to suggest new attribute subsets with an assessment criterion that scores different attributes^35^. The most suitable algorithm is the one that tests every possible subset of attributes and finds the best subset that minimizes the rate of error. However, this exhaustive search approach becomes computationally intractable in scenarios with more extensive feature spaces. The choice of evaluation metrics significantly affects the function. Various feature selection algorithms have been used, for example. ReliefF (RLF), correlation (CR), and OneR (ONR). As explained below, each feature selection algorithm has its own set of features:

#### ReliefF

The algorithmn allocates suitable weight to each attribute using an instance-based learning approach. The values of the class are distinguished based on the feature’s weight. These weights define feature rank, and those that attain a specific threshold are hand-picked to construct the final subset^36^. The algorithm operates by randomly choosing examples from the training dataset. For each sample instance, the closest example of the same class (nearest hit) and opposite class (nearest miss) is found^37^. It modifies a feature’s weight according to how nicely feature values differentiate the selected instance from its nearest miss and nearest hit. A feature would be assigned a higher weight if it distinguishes among examples from different classes and has an identical value for examples of the same class. The formula for weight updation in RLF is given below:

1 $$\begin{aligned} W_{y}=W_{y}-\frac{{\text {diff}}(Y, R, H)^{2}}{n}+\frac{{\text {diff}}(Y, R, M)^{2}}{n} \end{aligned}$$

Where Wy symbolizes the weight for feature Y, R is a randomly sampled example, H, M represents the closest hit, closest miss, and n describes the number of randomly sampled examples. The method diff() calculates the difference between two examples of a given feature. For nominal features, it is represented as 0 if the values are the same and 1 if the values are different. However, for continuous features, the actual difference is standardized to the interval $\{0,1\}$. Dividing the equation by n ensures weights in the interval $\{-1,1\}$. RLF is sensitive to feature interactions and tries to evaluate the probability change for the weight of the attribute Y as defined in Eq. (2).

2 $$\begin{aligned} \begin{aligned} W_y&= P\left( \frac{different\; value\; of\; Y }{ closest\;example \;of\; different \;class}\right) \\&\quad -P\left( \frac{different\; value\; of\; Y }{ closest \;example\; of\; same \;class}\right) \end{aligned} \end{aligned}$$

3 $$\begin{aligned} \begin{aligned} ReliefF_Y&=P\left( \frac{different\; value\; of\; Y }{ different\; class}\right) \\&\quad -P\left( \frac{different \;value\; of\; Y }{ same\; class}\right) \end{aligned} \end{aligned}$$

#### Correlation

It is an algorithm that uses the filter method to select features. It uses a heuristic-based method, which measures the effectiveness of individual features to predict the class label along with the level of inter-correlation between them^38^. The attributes with lesser correlation should be avoided, along with redundant attributes, as they may highly correlate with one or many of the remaining attributes. The formula used to filter out the redundant, irrelevant attributes, which contribute to the poor class prediction, is given in the equation as under:

4 $$\begin{aligned} M_{P}=\frac{j\overline{r_{cf}}}{\sqrt{j+j(j-1)\overline{r_{ff}}}} \end{aligned}$$

where $M_P$ represents the heuristic merit of a feature subset *P* having *j* attributes, $\overline{r_{cf}}$ is the mean attribute-class CR, and $\overline{r_{ff}}$ is the average attribute-attribute inter-correlation.

#### OneR

ONR is one of the simple classifiers in weka. The classifier is generally used for nominal data values. In this technique, OneR can produce a set of classification rules depending on the significance of a single feature^39^. The method selects the feature with the least error rate as its “one rule”^40^. The number of instances that do not belong to the majority class of the related feature value contributes to the error rate. It helps produce a baseline for classification performance and can deliver more satisfactory results than many other refined approaches^30^.

### Machine learning classifiers

In the following, we describe different classifiers like Support Vector Machine (SVM), Linear Discriminant Analysis (LDA), Logistic Regression (LR), and Multi-Layer Perceptron (MLP) used for training our model in this study.

#### SVM

The SVM is a classifier that helps in multi-class classification problems. It draws a hyperplane that maximizes the separation margin between two classes and minimizes the error^41^. The model provides significant advantages such as the absence of local minimums, sufficient generalization to the new objects, and a representation that relies on a few parameters^42^. Given a training set of input vectors ${\textbf{x}}_{i} \in R^{d}$, $i=\{1,\ldots ,N_{t}\}$ for d dimensional input space and outputs $y_{i} \in \{1,-1\}$. The SVM hyperplane Eq. (5) is given as under:

5 $$\begin{aligned} y_{i}&= sign({\textbf{w}} \cdot \textbf{xy}^T_{i} + b) \end{aligned}$$

where ${\textbf{x}}$ and ${\textbf{w}}$ represent input and constant vectors in the hyperplane, respectively. While the training input vector ${\textbf{x}}_{i}$ represents the features and *sign*() is a signum function with ±1 output. The objective is to minimise Eq. (6).

6 $$\begin{aligned}&\min _{w,b,\zeta } \, \frac{1}{2} ||{\textbf{w}}||^{2}+C_{b}\sum \zeta _{i}\nonumber \\ \text { (subject to) } \text { }&y_{i} ({\textbf{w}}^T{\textbf{x}}_{i}+b) \ge 1-\zeta _{i}&(\forall i) \nonumber \\&\zeta _{i} \ge 0&(\forall i) \end{aligned}$$

where $\zeta _{i}$ penalises objective function for data samples that cross margins meant for that particular class and $C_{b}$ is the box constraint.

#### Linear discrimination analysis

The classifier is used for preprocessing in machine learning applications, pattern classification, and LDA. The purpose of the model is to minimize lower dimensional space with optimized class separability and minimize computational cost^43^.

#### Logistic regression

LR is a variation of the traditional regression approach. It is applied when the dependent variable is binary in nature^44^. Like other regression models, it is also a predictive analysis model, which interprets data and explains the association between one dependent variable and one or more nominal, ordinal independent variables. In this approach, the dependent variable is the probability that an event may occur; therefore, the resulting value has a discrete number of responses, restrained between 0 and 1. It can be shown as follows:

7 $$\begin{aligned} P(\vec {x})=\frac{1}{1+e^{-f(\vec {x})}}=\frac{e^{f(\vec {x})}}{1+e^{f(\vec {x})}} \end{aligned}$$

Where $P(\vec {x})$ is the probalility of a specific output event, $x_1,x_2,\ldots ,x_n$ is an input vector equal to the independent predictors or variables, and $f(\vec {x})$ is the LR prototype.

#### Multi-layer perceptron

**Figure 2:**
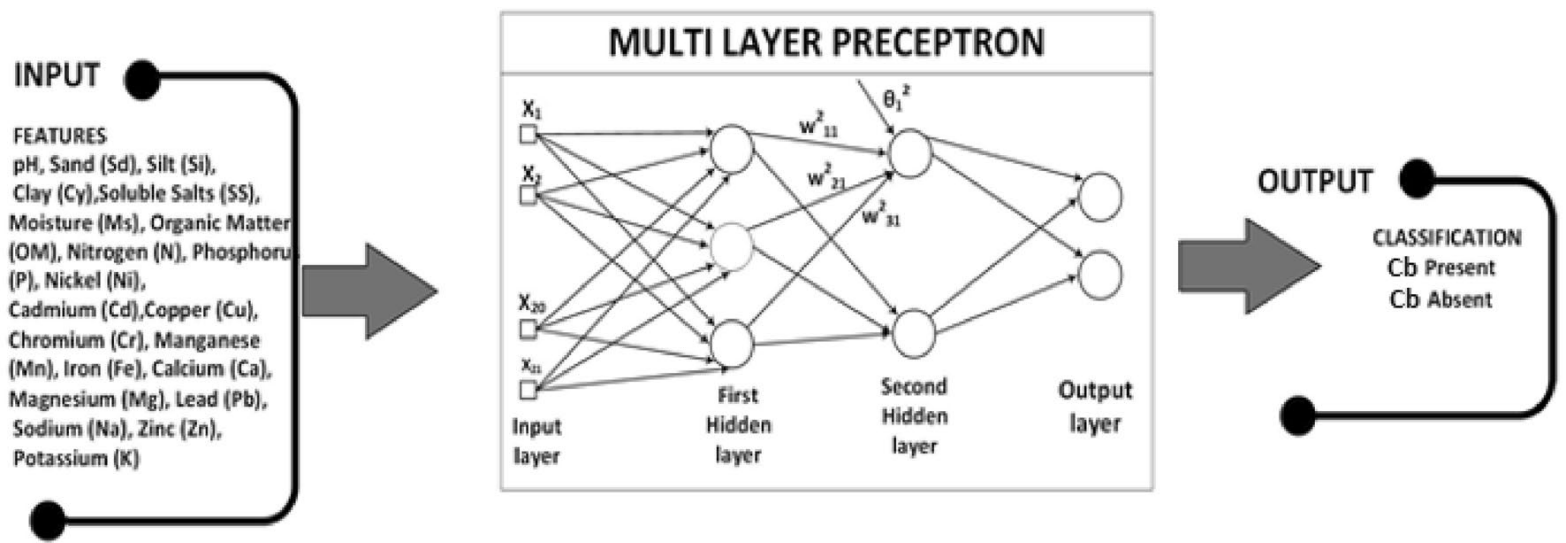
MLP model with inputs $\{X1,\ldots ,X21\}$, two outputs and two hidden layers with three and two hidden units in each layer, respectively.

MLP is a complement of a feed-forward neural network. It comprises three kinds of layers—an input, output, and a hidden layer, as illustrated in Fig. 2. The input layer acquires the input data for processing. The out layer performs the essential task of classification and prediction. A number of hidden layers are the real computation engine of the design, which reside between the input and output layer of the MLP. An MLP uses backpropagation, a technique through which the weights in a neural network are optimized. The MLP approximates any continuous function and resolves tasks that are not linearly separable. It usually performs recognition, pattern classification, approximation, and prediction tasks. The calculations taking place at each neuron in the output and hidden layer are as under:

8 $$\begin{aligned} \textrm{O}(\textrm{y})&= \mathrm {s_2}(\textrm{B}(2)+\textrm{W}(2) \textrm{h}(\textrm{y})) \end{aligned}$$

9 $$\begin{aligned} \textrm{h}(\textrm{y})&= \Phi (\textrm{y})=\mathrm {s_1}(\textrm{B}(1)+\textrm{W}(1) \textrm{y}) \end{aligned}$$

Where $\{W(1), W(2)\}$ and $\{B(1), B(2)\}$ represent weights and biases of the pervious and next layer. *y* reperensts the output of pervious layer and inner vector probuct of Y with the weights of the current layer *W*(1) is computed, a bias vector *B*(1) is added and the result is used as an input for the activation function $s_1()$ . The activation functions are $\{s_1, s_2\}$. Usually the activation functions that are used are tanh and sigmoid, represented as $\tanh (a)=\left( e^{a}-e^{-a}\right) /\left( e^{a}+e^{-a}\right)$ and ${\text {sigmoid}}(a)=1 /\left( 1+e^{-a}\right)$, respectively.

## Experiments

### Data description

The experiments are conducted using the *C. burnetii* soil feature dataset, consisting of 94 specimens. Each specimen comprises 21 soil features. We need a supervised dataset to formulate a predictive model using classification techniques. So, the next step is to allocate suitable labels to every instance in the dataset. Thus, for +Ve and -Ve *C. burnetii* soil samples, class labels “1” and “0” were assigned, respectively.

### Software tools

Weka is employed to train and test the *C. burnetii* dataset on various soil features^45^. First, we saved the details of the soil attribute dataset for *C. burnetii* in a CSV file and then opened the file in Weka’s GUI interface. Second, we ranked these soil features using various feature selection methods. Third, we selected a classification algorithm and then calculated its accuracy by selecting top-ranked attributes one by one from the list using a nested subset approach. For some classifiers, Matlab libraries are employed during experimentation.

### Performance evaluation

The soil dataset is utilized to test and train the model using various machine learning classifiers by applying a 10-fold cross-validation approach. The approach randomly divides the dataset into ten subsets of the same size, where each part has nearly an identical class distribution. Each subset is applied one by one as a test dataset, while the remaining subsets of the split serve as a training set. At each step, the model’s accuracy is calculated, and the results of all outcomes are averaged to generate the final accuracy.

## Results

The current section presents the experimental results of the features-ranking models and compares their performance against different machine learning classifiers. Various algorithms are used for classification: SVM, LDA, LR, and MLP. A 10-folds cross-validation is applied to access the performance better and avoid overfitting.

Firstly, the features of the *C. burnetii* dataset are ranked using three feature-ranking models. Table 3 illustrates the ranking for different feature-ranking algorithms, like CR, ONR, and RLF. The column “Attribute Index” displays a unique index value for every attribute, where pH has an index value=1, moisture (MO)=2, soluble salts (SS)=3, and so on. The first row in columns rk(CR), rk(ONR), and rk(RLF) shows the top ranked attributes 8, i.e.(N), 17 ,i.e.(Cd), and 12, i.e.(Cr), respectively. The second row shows the following top ranked attributes 4, i.e.(OM), 21, i.e.(K), and 21, i.e.(K), respectively. Similarly, the last row shows last of the top ranked attributes 14, i.e.(Mn), 20, i.e.(Ca), 14, i.e.(Mn), respectively. Moreover, if we assess the top 11 features from all the feature-ranking methods in Table 3, the following conclusions can be drawn:

1. 6 features i.e.{OM, N, Ni, Cr, Cd, K} are recurring for all ranking methods.
2. 7 features i.e.{ Si, OM, N, Ni, Cr, Cd, K} are similar between ONR and RLF.
3. 8 features i.e.{ pH, SS, OM, N, Ni, Cr, Cd, K} are similar between ONR and CR.
4. 9 features i.e.{ OM, N, Ni, Na, Mg, Cr, Cd, Ca, K} are similar between CR and RLF.

Similarly, Table 3 shows that out of the 9 least-significant features, 6 features, i.e.$\{\hbox {Pb},\,\hbox {MO},\,\hbox {P},\,\hbox {Cu},\,\hbox {Mn},\,\hbox {cy}\}$, are recurring among all ranking methods.

**Table 3:** Attribute-ranking for *C. burnetii* soil attribute dataset by different Feature-ranking techniques.

Attribute index	Soil attributes	rk(CR)	rk(ONR)	rk(RLF)
1	pH	8	17	12
2	Moisture (MO)	4	21	21
3	Soluble Salts (SS)	3	1	18
4	Organic Matter (OM)	21	12	8
5	Clay (cy)	18	15	4
6	Sand	1	13	17
7	Silt (Si)	17	3	20
8	Nitrogen (N)	12	7	10
9	Phosphorus (P)	10	8	19
10	Magnesium (Mg)	13	6	7
11	Copper (Cu)	20	4	13
12	Chromium (Cr)	2	10	2
13	Nickel (Ni)	19	2	3
14	Manganese (Mn)	7	14	15
15	Cobalt (Co)	5	19	6
16	Lead (Pb)	11	9	16
17	Cadmium (Cd)	6	18	1
18	Sodium (Na)	16	11	9
19	Iron (Fe)	15	16	11
20	Calcium (Ca)	9	5	5
21	Potassium (K)	14	20	14

Secondly, we perform a two-phase feature ranking to determine the contribution of each attribute toward the persistence of *C. burnetii* in soil. Initially, attributes are ranked based on feature-ranking methods, and then a combination of techniques is applied to calculate the weighted score to determine the final soil attribute rank. The top-ranked and least-ranked attributes are displayed separately in Tables 4 and  5. These tables show each feature ranking method’s scores and the final aggregate score of each soil attribute for the *C. burnetii* dataset. The aggregate score is the sum of the scores of all the attribute ranking methods. If the aggregate score is on the lower side, higher would be the rank of an attribute. Similarly, if the score is on the higher side, the lower would be the rank of the attribute.

**Figure 3:**
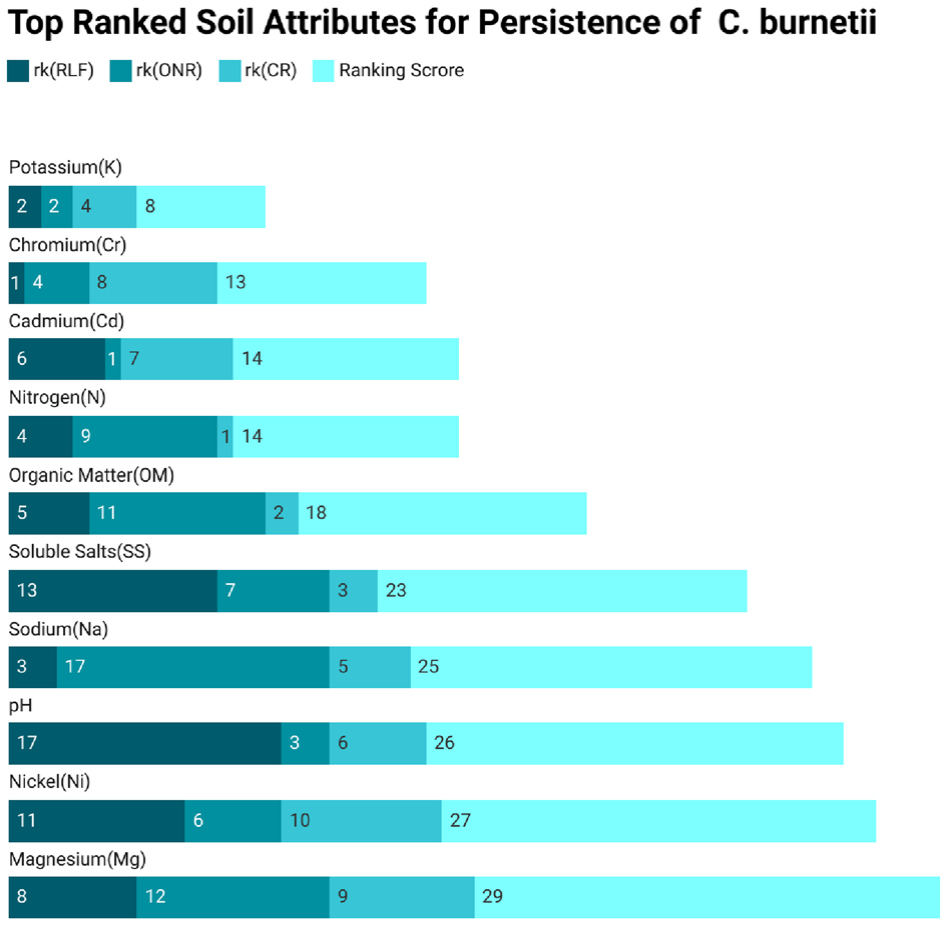
Individual and Aggregate Score of Top Ranked attributes of *C. burnetii* Soil dataset using Feature-ranking methods.

**Figure 4:**
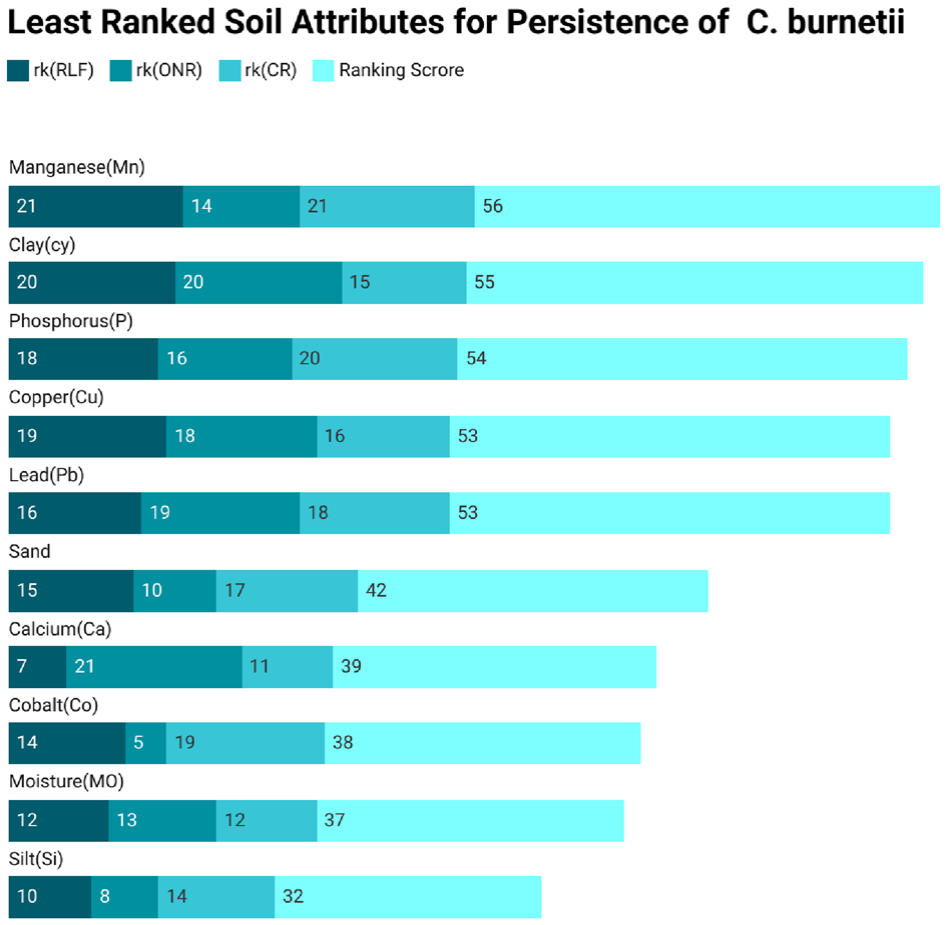
Individual and Aggregate Score of Least Ranked attributes of *C. burnetii* Soil dataset using Feature-ranking methods.

The first row depicts $\{\hbox {K}\}$ ranked 2nd by RLF and ONR, 4th by CR, and the last column shows its aggregate score of 8, which is the sum of scores of all the attribute ranking methods, i.e.(2 + 2 + 4 = 8). The second row shows that $\{\hbox {Cr}\}$ is ranked 1, 4, and 8 by RLF, ONR, and CR, respectively, with an aggregate score of 13. Similarly, the last row shows that $\{\hbox {Mg}\}$ is ranked 8, 12, and 9 by RLF, ONR, and CR, respectively, with an aggregate score of 29. Now $\{\hbox {K}\}$ is the top ranked attribute, as its aggregate score, i.e.(8) is minimum, $\{\hbox {Cr}\}$ $2^{\textrm{nd}}$ top ranked attribute with an aggregate score of 13. Similarly, the results in the Table  5 shows that $\{\hbox {Mn}\}$ is the least ranked attribute with an aggregate score of 56, which is the sum of scores of all the attribute ranking methods, i.e.(21+14+21=56) and, then comes $\{\hbox {cy}\}$, $\{\hbox {P}\}$, and $\{\hbox {Cu}\}$ with aggregate scores of 55, i.e.(20+20+15), 54, i.e.(18+16+20), and 53, i.e.(19+18+16), respectively, and so on. The stacked bar chart further elaborates the picture by showing the score of feature ranking of different methods and the aggregate score for each attribute in different color schemes. These charts in Figs. 3 and  4 display the top and least ranked features, where rk(RLF), rk(ONR), and rk(Cr) in various flavors of blue represent the ranking score for RLF, ONR, and CR. Similarly, the Ranking Score symbolizes the sum of scores of all the attribute ranking methods for an attribute, which is represented in light blue.

**Table 4:** List of Top Ranked Attributes for *C. burnetii* soil attribute dataset.

Top ranked attributes	rk(RLF)	rk(ONR)	rk(CR)	Ranking score of each attribute
Potassium (K)	2	2	4	8
Chromium (Cr)	1	4	8	13
Cadmium (Cd)	6	1	7	14
Nirogen (N)	4	9	1	14
Organic Matter (OM)	5	11	2	18
Soluble Salts (SS)	13	7	3	23
Sodium (Na)	3	17	5	25
pH	17	3	6	26
Nickel (Ni)	11	6	10	27
Magnesium (Mg)	8	12	9	29

**Table 5:** List of Least Ranked Attributes for *C. burnetii* soil attribute dataset.

Least ranked attributes	rk(RLF)	rk(ONR)	rk(CR)	Ranking score of each attribute
Manganese (Mn)	21	14	21	56
Clay (cy)	20	20	15	55
Phosphorus (P)	18	16	20	54
Copper (Cu)	19	18	16	53
Lead (Pb)	16	19	18	53
Sand	15	10	17	42
Calcium (Ca)	7	21	11	39
Cobalt (Co)	14	5	19	38
Moisture (MO)	12	13	12	37
Silt (Si)	10	8	14	32

The top-ranked features shown in Fig. 3 reflect that Potassium (K) is the most significant attribute, where K is ranked $2^{\textrm{nd}}$ by RLF and ONR, $4^{\textrm{th}}$ by CR, so its aggregate score is 8, which is the sum of scores of all the attribute ranking methods, i.e.(2+2+4=8). Similarly, the least-ranked features are shown in Fig. 4, which portrays that Mn is the least significant attribute with a ranking score of 56, which is the sum of individual feature scores of 16, 21, and 20 for RLF, ONR, and CR, respectively.

**Table 6:** A Comparison of results from various Feature-ranking methods against different Machine learning classifiers using *C. burnetii* dataset.

		Subset																				
FRM	Clf	1	2	3	4	5	6	7	8	9	10	11	12	13	14	15	16	17	18	19	20	21
CR	rk	8	4	3	21	18	1	17	12	10	13	20	2	19	7	5	11	6	16	15	9	14
	SVM	64.89	62.77	61.7	62.77	61.7	64.89	67.02	67.02	68.09	71.28	70.21	70.12	75.53	82.98	76.6	76.6	75.53	73.4	74.47	74.47	73.4
	LDA	62.8	61.7	63.8	64.9	62.8	61.7	62.8	64.9	67	63.8	69.1	68.1	78.7	78.7	78.7	76.6	74.5	75.5	74.5	73.4	73.4
	LR	62.77	61.7	62.77	63.83	60.64	59.57	57.45	59.57	63.83	65.96	67.02	67.02	71.28	73.4	75.53	74.47	71.28	70.21	70.21	69.14	69.14
	MLP	60.64	59.57	62.77	58.51	60.64	56.38	60.64	65.96	62.77	62.77	69.15	69.15	75.53	79.79	75.53	78.72	74.47	74.47	74.47	71.28	73.4
RLF	rk	12	21	18	8	4	17	20	10	19	7	13	2	3	15	6	16	1	9	11	5	14
	SVM	57.45	53.19	56.38	62.77	61.7	64.89	69.15	72.34	75.53	78.72	80.85	81.91	81.91	79.79	75.53	75.53	76.6	75.53	74.47	74.47	73.4
	LDA	55.3	52.1	57.4	64.9	63.8	69.1	70.2	71.3	78.7	77.7	78.7	77.7	79.8	78.7	75.5	76.6	76.6	74.5	75.5	73.4	73.4
	LR	57.45	47.87	59.57	62.77	62.77	64.89	68.09	75.73	76.6	79.79	78.72	77.66	74.47	74.47	73.4	71.28	71.28	69.14	70.21	69.14	69.14
	MLP	55.32	53.19	58.51	57.45	61.7	68.09	73.4	78.72	75.53	77.66	77.66	80.85	78.72	75.53	74.47	77.66	75.53	76.6	73.4	71.28	73.4
ONR	rk	17	21	1	12	15	13	3	7	8	6	4	10	2	14	19	9	18	11	16	5	20
	SVM	57.45	56.38	57.45	64.89	62.77	65.96	70.21	72.34	71.28	71.28	70.21	72.34	71.28	71.28	73.4	74.47	74.47	73.4	75.53	75.53	73.4
	LDA	59.6	54.3	64.9	64.9	69.1	64.9	70.2	68.1	70.2	70.2	69.1	66	66	68.1	71.3	72.3	70.2	71.3	70.2	64.9	73.4
	LR	60.64	54.25	63.83	64.89	68.09	63.83	68.09	65.96	71.28	72.34	71.28	70.21	68.09	64.89	69.15	65.96	65.96	68.09	67.02	64.89	69.14
	MLP	59.57	54.26	61.7	63.83	64.89	62.77	69.15	65.96	72.34	71.28	76.6	65.96	68.09	67.02	69.15	70.21	67.02	68.09	67.02	71.28	73.4

Thirdly, we evaluated the performance of these feature-ranking methods to different machine learning classifiers. The result of the experiments is shown in Table 6. For every feature-ranking technique, the row “rk” illustrates the ranking sequence of attributes. Then the table presents the results of classifiers ( MLP, LR, LDA, and SVM) according to the ranking sequence of each feature-ranking model. The accuracy ranges from 82.98% (SVM) to 53.19% (SVM) while applying various ranking models and classification techniques. The most relevant feature for CR is $\{\hbox {N}\}$. Using this feature, SVM, LDA, and LR produce a classification accuracy of 63.91%, 62.9%, and 62.89% for CR, respectively. The most relevant feature for ONR is $\{\hbox {Cd}\}$, and RLF is $\{\hbox {Cr}\}$. Using Cd(ONR), LDA generated an accuracy of 59.6%, and Cr(RLF), SVM produces an accuracy of 57.45%. We can infer various conclusions from the analysis of Table 6: (a) The three attribute-ranking models deliver distinct rankings, which generate different classification outcomes. (b) The pair of $\{\hbox {CR}+\hbox {SVM}\}$ gives best classification accuracy of (82.98%) for only 14 soil features. (c) In principle, the order of best classification performance is arbitrary: (CR+SVM,82.98%), (RLF+SVM,81.91%), (ONR+SVM,75.53%), (CR+LDA,78.7%), (RLF+LDA,79.8%), (ONR+LDA,73.4%), (CR+LR,75.53%), (RLF+LR,79.79%),(ONR+LR,72.34%), (CR+MLP,79.79%), (RLF+MLP,80.85%), (ONR+MLP,76.6%). (d) Results show that machine learning classifiers like LDA, LR, and MLP showed better accuracy using the RLF feature-ranking than other feature ranking approaches. (e) CR stands next to RLF and produces better classification results for SVM than other ranking methods. (f) MLP performs better than other machine learning classifiers for ONR feature ranking. (g) The 14 soil attributes for which $\{\hbox {CR}+\hbox {SVM}\}$ generates the best classification accuracy are $\{\text {N,OM,SS,K,Na,pH,Cd,Cr,Mg,Ni,Ca,MO,Fe,Si}\}$. In contrast, the other models, like $\{\hbox {RLF}+\hbox {SVM}\}$ and $\{\hbox {RLF}+\hbox {MLP}\}$ utilize 12 soil features to generate their best classification accuracies of 81.91% and 80.85%, respectively.

**Figure 5:**
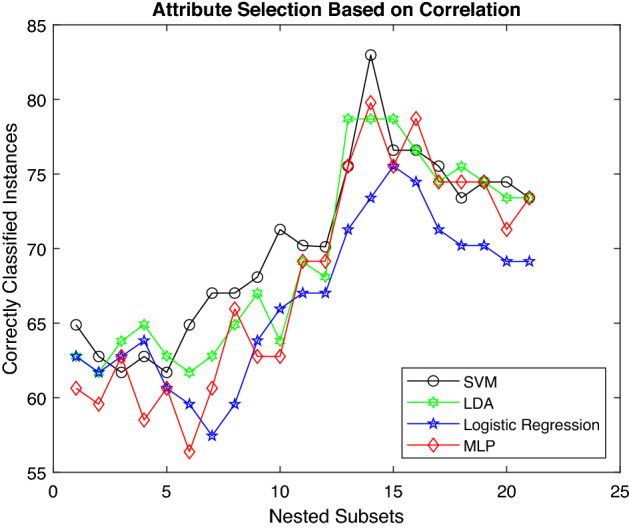
Accuracy of various classifiers depending upon CR.

**Figure 6:**
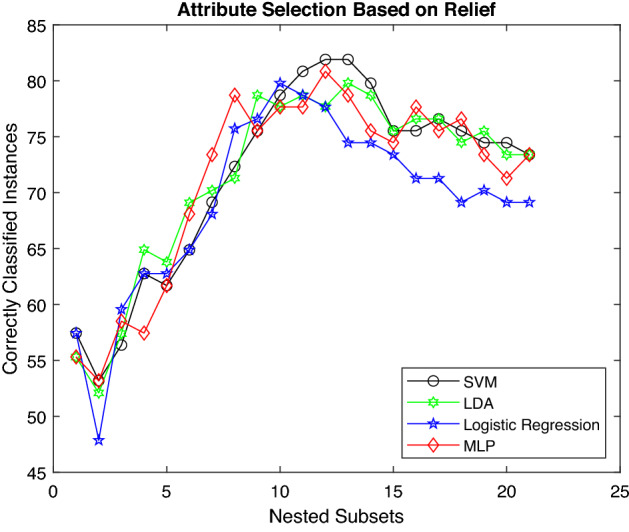
Accuracy of various classifiers depending upon RLF.

**Figure 7:**
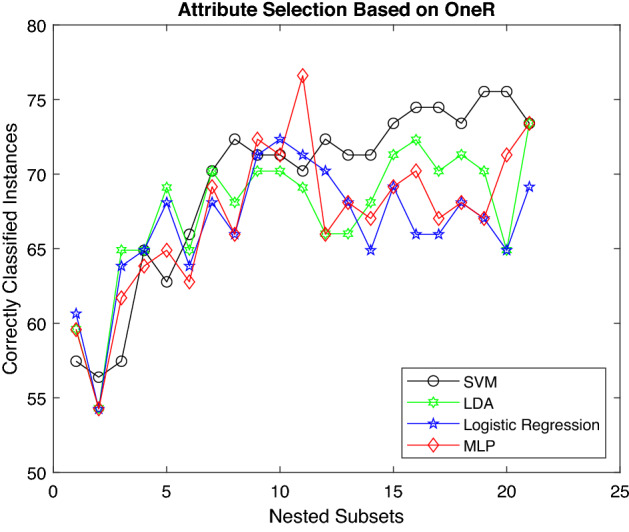
Accuracy of various classifiers depending upon ONR.

Figures 5, 6 and 7 demonstrate the change in accuracy of machine learning classifiers as the number of soil features is varied while applying feature–ranking approaches. Figure 5 shows the accuracy of machine learning models using CR as attribute–ranking technique. Although the feature subset is similar, LDA performance is better than other classifiers for initial-level features. However, SVM shows excellent results for mid-level features. All the classifiers display a considerable decrease in accuracy for the last set of features. The results show that SVM generates a classification accuracy of 82.98%, which is far better than other models. So, the overall performance of SVM is far better than other machine learning classifiers.

Figure 6 represents accuracy curves for classification algorithms using the RLF feature–ranking technique. Although all the classifiers show a similar trend, SVM and MLP achieve a classification accuracy of 81.91% and 80.55% higher than any other classification method. All the classifiers shows similar trend for initial set of features. However, LDA and MLP seem to perform better than other classifiers. But, for mid-level features, LDA and MLP stand close to SVM. Nevertheless, the overall performance of SVM is better than other classifiers.

Figure 7 illustrates the accuracy of classification models using ONR as an attribute-ranking technique. However, all the classifiers show a similar trend for a nested subset of soil features except MLP, which shows a sharp increase for mid-level features. Although LR and LDA show better results for the initial features, SVM outperforms other classifiers for the last subset of features.

In summary, the results propose that: (a) 6 features that significantly contribute towards the persistence of the pathogen in the environment are $\{\hbox {K},\,\hbox {Cr},\,\hbox {Cd},\,\hbox {N},\,\hbox {OM},\,\hbox {SS}\}$ (b) 5 least contributing features for Coxiella are $\{\hbox {Mn},\hbox {cy},\,\hbox {P},\,\hbox {Cu},\,\hbox {Pb}\}$. c) Feature ranking using RLF generates better results for all machine learning algorithms than other feature-ranking models. (d) The classification results of SVM surpass all other machine learning classifiers. (e) $\{\hbox {CR}+\hbox {SVM}\}$ produces the best accuracy of 82.98% for the initial 14 soil features $\{\text {N,OM,SS,K,Na,pH,Cd,Cr,Mg,Ni,Ca,MO,Fe,Si}\}$. (f) in multi-dimensional classifications, various machine learning models depict a similar trend. Therefore, the correct classifier selection is essential to yielding good classification results in unseen examples.

### Comparision with previous machine learning approaches

Although some researchers applied machine learning to classify soil-borne pathogens like *F. tularensis* and the environment that help its persistence in soil, there needs to be more data available specifically for *C. burnetiia* in the soil-related environment, as shown in Table  7. Furthermore, our model applies a two-phase feature-ranking on a novel *C. burnetiia* dataset, contrary to previous works.

**Table 7:** Comparison with previous Machine learning approches.

Approach	Soil pathogen	Novel dataset	Classification & feature-ranking	Two-phase feature-ranking	Most relavant features	Least contributing features	Classification accuracy (%)
Our Model	*C. burnetiia*	$\checkmark$	$\checkmark$	$\checkmark$	Potassium, nitrogen, organic matter, chromium, cadmium, and magnesium	Manganese, phosphorous, clay, moisture, and copper	82.98
Ahmad et al.^30^	*F. tularensis*	$\times$	$\checkmark$	$\times$	Clay, nitrogen, organic matter, soluble salts, zinc, silt and nickel	Potassium, phosphorous, iron, calcium, copper, chromium and sand	84.35
shahbaz et al.^29^	*F. tularensis*	$\times$	$\times$	$\times$	$\times$	$\times$	82.61

### Discussions

Machine learning models are used as a standard in different disciplines, for example, soil classification^46^, medical science^47^, bio-informatics^48^, and agriculture^49–51^. Our research reveals that machine learning models, instead of contemporary statistical models show exceptional results for classifying *C. burnetii* and understanding the pathogen’s behavior in the soil-related environment.

The results propose that potassium, chromium, cadmium, nitrogen, organic matter, and soluble salts are the top 6 most significant features for the persistence of the Coxiella, as exhibited in Table 4. Previous works also propose that abiotic characteristics such as pH, organic matter, and soil nutrients, are not only the driving force for soil bacterial community^52–55^ but are also positively linked with the persistence of soil pathogens^56–58^. Some recent studies^9,22,23^ also highlight the significance of soil’s physiochemical characteristics, like organic matter, soluble salt, nitrogen, clay, potassium, cobalt, chromium, and cadmium, etc., for the sustenance of B. anthracis, *C. burnetii* and *F. tularensis*.

Our analysis further reveals that potassium is the most noteworthy feature for the presence of Coxiella in soil. Some fantastic works^9,22,23,59^ prove that the prevalence of various pathogens like B. anthracis, *C. burnetii*, and *F. tularensis* positively correlates to potassium in the soil. The next essential features that improve the likelihood of persistence of the pathogenic bacteria are chromium, cadmium, nitrogen, organic matter, and soluble salts. Studies^23,56–58^ in the recent past indicate that the presence of organic matter and chromium is helpful for persistence of pathogens in the soil. Another study^60^ reveals that nitrogen is essential for the sustenance of pathogens within their plant and animal hosts. A study^22,61^ suggests that cadmium, nitrogen, soluble salts and organic matter positively correlate with the prevalence of *F. tularensis* in soil. The prevalence of B. anthracis is also associated with the presence of organic matter, chromium, and potassium in soil^23^. Recent works^9,59^ highlight that soluble salts is positively correlated with the persences of *C. burnetii* and *F. tularensis*. Similarly, a work^61^ provides evidence that nitrogen and organic matter are helpful in the persistance of *C. burnetii* and another research also illustrates that nitrogen and organic matter are also positively related to the sustenance of a nitrogen-fixing bacteria called A. brasilense^62^.

The remaining contributing features from Table 4 are sodium, pH, nickel, and magnesium. Previous researches^53–55^ show that soil texture, pH, and nutrients are essential for bacterial communities. Our results conform with a recent study^23^ that reveals that features like magnesium, potassium, and sodium are positively correlated to *C. burnetii* in soil-related environments. Another work^9^ also shows a substantial difference between Coxiella negative and positive sites with reference to magnesium and sodium. A study^30^ also reveals that *F. tularensis* has a positive affinity with souble salts, nickel, and pH for its existence in soil. Another research^59^ reveals that soluble salts and nickel positively contribute towards the presence of *F. tularensis*. Magnesium plays a substantial part in the persistence of microbes during starvation and cold shocks^63^. A work^25^ illustrates magnesium, sodium, potassium, and sulfate are conducive to *F. tularensis* growth in soil and water.

Our study also depicts that silt, moisture, and cobalt fall in the middle. Previous research reveals that silt possesses substantial organic matter due to the rise in surface area compared to the sandy portion, which may augment the possibility of the prevalence of pathogens^64^. Another research^23^ shows that the persistence of *C. burnetii* is associated with higher concentration of cobalt in the enviornment. A study^22^ reveals that the persistence of *F. tularensis* is positively correlated to the presence of silt in soil. Another work^65^ proposes that *F. tularensis* has a great affinity to moisture and low temperature.

Our machine learning analysis reveals that the least contributing seven features are manganese, clay, phosphorous, copper, lead, sand, and calcium as shown in Table 5. A recent research^9^ also substantiates our viewpoint by exhibiting no significant difference between Coxiella negative and positive sites regarding manganese, phosphorous, clay, lead, copper, and sand in the soil. A study^22^ also reveals that manganese, phosphorous, calcium, copper, and sand do not show any positive affinity with *F. tularensis* in soil. Similar research^59^ also reveals that clay, phosphorous, copper, lead, sand, and calcium are not positively correlated with *F. tularensis*. Some suggest^66^ that during hot and dry weather, high manganese contents are seen in *B. pseudomallei* positive sites as appose to negative sites. However, others^67^ believe that the aerobic heterotrophic population of microbes is very susceptible to different minerals, like cadmium, nickel, manganese, mercury, chromium, copper, and zinc. An analysis also reveals that manganese and zinc are essential for biological processes, and they exist as protein components in many species^67,68^. Some works propose that zinc helps in multiple cellular functions, like pH regulation, metabolism, bacterial gene expression, DNA replication, glycolysis, synthesis of Amino acids, and processes as a cofactor of microbial virulence^69^. However, the surplus amount of zinc can cause toxicity; thus, these microbes possess a mild structure to maintain zinc’s equilibrium for executing crucial cellular functions and abstain from the damages it may cause^70^.

Classification outcomes of *C. burnetii* in soil employing different machine learning techniques reveal that SVM surpasses all other machine learning models by generating an accuracy of 82.98% utilizing the initial 14 top-ranked features.

## Conclusion

The soil texture, physical and chemical factors play an important role in the growth and survival of bacteria. Thus, their relationship with *C. burnetii* is investigated in this study. The recent machine learning models can help us better understand the association of microbes with various soil features. The research presents the classification and feature-ranking of the pathogen using a soil feature dataset. Potassium is the top-ranked attribute, followed by chromium, cadmium, nitrogen, and organic matter. However, manganese, clay, phosphorus, and copper are the least contributing features. The RLF shows the best result for most of the ranking algorithms. SVM produces the best accuracy of 82.98% for the initial 14 soil features $\{\hbox {N},\hbox {OM},\hbox {SS},\hbox {K},\hbox {Na},\hbox {pH},\hbox {Cd},\hbox {Cr},\hbox {Mg},\hbox {Ni},\hbox {Ca},\hbox {MO},\hbox {Fe},\hbox {Si}\}$, using CR. In contrast, like SVM and MLP generate accuracies of 81.91%, and 80.85%, respectively for RLF. These machine learning models can also help us better understand the contribution of various soil features towards the survival of the pathogenic bacteria in the environment.

## Future works

Various pathogens behave differently in the environment due to variations in their cell structure. Some of these pathogens are highly resistant to environmental factors and can survive in the environment for years. Understanding how these pathogens behave in different environmental conditions is crucial for the research community to predict future outbreaks. So machine learning models can significantly help in achieving this task. In our previous works, we tried to classify and learn how *F. tularensis* behaves in the environment. Our current work focuses on the classification of *C. burnetii* and how it behaves in the environment. In the future, we intend to expand this work for other pathogens to devise a comprehensive model that could help us in predicting various disease outbreaks by these pathogens.

## Data Availability

The corresponding author can be contacted at fareed.ahmad@uvas.edu.pk for data relating to this study.

## Abbreviations

Ca: Calcium
Cb: Coxiella burnetii
Cd: Cadmium
Co: Cobalt
Cr: Chromium
CR: Correlation
Cu: Copper
cy: Clay
Fe: Iron
Ft: *Francisella tularensis*
K: Potassium
LDA: Linear discriminant analysis
LR: Logistic regression
Mg: Magnesium
MLP: Multi-layer perceptron
Mn: Manganese
MO: Moisture
N: Nitrogen
Na: Sodium
Ni: Nickel
OM: Organic matter
ONR: OneR
P: Phosphorus
Pb: Lead
RLF: ReliefF
Si: Silt
SS: Soluble salt
SVM: Support vector machine
